# Use of Alcohol and Addictive Drugs During the COVID-19 Outbreak in Norway: Associations With Mental Health and Pandemic-Related Problems

**DOI:** 10.3389/fpubh.2021.667729

**Published:** 2021-06-14

**Authors:** Tore Bonsaksen, Øivind Ekeberg, Inger Schou-Bredal, Laila Skogstad, Trond Heir, Tine K. Grimholt

**Affiliations:** ^1^Department of Health and Nursing Sciences, Faculty of Social and Health Sciences, Inland Norway University of Applied Sciences, Elverum, Norway; ^2^Faculty of Health Studies, VID Specialized University, Sandnes, Norway; ^3^Psychosomatic and Consultation-Liaison Psychiatry, Division of Mental Health and Addiction, Oslo University Hospital, Oslo, Norway; ^4^Faculty of Medicine, University of Oslo, Oslo, Norway; ^5^Department for Cancer, Oslo University Hospital, Oslo, Norway; ^6^Department of Research, Sunnaas Rehabilitation Hospital HF, Bjørnemyr, Norway; ^7^Department of Nursing and Health Promotion, Faculty of Health Sciences, Oslo Metropolitan University, Oslo, Norway; ^8^Norwegian Center for Violence and Traumatic Stress Studies, Oslo, Norway; ^9^Institute of Clinical Medicine, University of Oslo, Oslo, Norway; ^10^Faculty of Health Studies, VID Specialized University, Oslo, Norway; ^11^Department of Acute Medicine, Oslo University Hospital, Oslo, Norway

**Keywords:** alcohol, COVID-19, pandemic, population survey, substance use

## Abstract

**Background:** The outbreak of COVID-19 has had a major impact on people's daily life. This study aimed to examine use of alcohol and addictive drugs during the COVID-19 outbreak in Norway and examine their association with mental health problems and problems related to the pandemic.

**Methods:** A sample of 4,527 persons responded to the survey. Use of alcohol and addictive drugs were cross-tabulated with sociodemographic variables, mental health problems, and problems related to COVID-19. Logistic regression analyses were used to examine the strength of the associations.

**Results:** Daily use of alcohol was associated with depression and expecting financial loss in relation to the COVID-19 outbreak. Use of cannabis was associated with expecting financial loss in relation to COVID-19. Use of sedatives was associated with anxiety, depression, and insomnia. Use of painkillers was associated with insomnia and self-reported risk of complications if contracting the coronavirus.

**Conclusion:** The occurrence of mental health problems is more important for an understanding of the use of alcohol and addictive drugs during the COVID-19 outbreak in Norway, compared to specific pandemic-related worries.

## Introduction

The outbreak of COVID-19 has had a major impact on people's daily life. To prevent the spread of the coronavirus disease, strict policies have emphasized keeping a physical distance to other people, commonly known as social distancing (1, 2). These policies introduced abrupt changes in economic life. Most travels and events were canceled, and non-vital businesses were closed. Consequently, financial problems suddenly became a reality for many businesses, many employees were temporarily furloughed from their jobs (3) and unemployment rates increased rapidly (4). During this time, there has been a worldwide growing concern that living under restrictive social distancing policies and a general sense of uncertainty may have a profound impact on the mental health of the population (5–8).

While the use of various pharmaceuticals generally reflects the burden of disease in a society, the use of alcohol and addictive drugs may also vary according to changes in environmental conditions. Thus, the use of these substances may be expected to rise during difficult times, such as the COVID-19 pandemic. Studies of healthcare personnel during previous infectious disease outbreaks have shown alcohol use to be higher among those who worked in high-risk locations and situations, compared to those who worked in low-risk situations (9, 10). Moreover, during the current COVID-19 crisis, evidence of increased alcohol use in the general population (11), as well as a higher demand for cannabis products on the darknet (12), have been found.

Recent studies based on data collected during the COVID-19 pandemic have demonstrated associations between higher alcohol use and middle age, higher income, job loss, stress, sleep problems, and depression (13). Thus, the use of alcohol may be associated with distal (sociodemographic factors, mental health) and proximal factors (circumstances evoked by the COVID-19 situation) alike. While mental health problems, such as depression and anxiety, have been linked with increased alcohol use during the pandemic (14), the consequences of the COVID-19 outbreak (e.g., fear of virus transmission, loneliness, and financial problems) may be related to increased use of alcohol and other substances. Thus, to increase the understanding of use of alcohol and addictive drugs in the COVID-19 context, the explanatory power of a wider range of variables related to the pandemic's consequences needs to be considered. However, we are unaware of similar population studies concerned with the use of multiple addictive drugs during the COVID-19 outbreak. In addition, while recent studies have examined the prevalence of use of alcohol and addictive drugs in the general Norwegian population (15), such studies may not reflect the use of such substances during extraordinary circumstances such as the current pandemic. The aims of the study were to examine use of alcohol and addictive drugs during the COVID-19 outbreak and examine their association with mental health problems and problems related to the pandemic.

## Materials and Methods

### Design

A population-based cross-sectional study that dealt with reactions in the Norwegian population after the COVID-19 outbreak (the CORONAPOP study) (16, 17), was conducted with a web-link open to all citizens between April 8th, 2020 and May 20th, 2020. The web-link was hosted and disseminated by several institutions, including Oslo University Hospital, Sunnaas Rehabilitation Hospital, University of Oslo, and Oslo Metropolitan University. The link to the survey was further disseminated on social media platforms, such as Facebook, Twitter, LinkedIn, and Instagram, by the individual researchers and other individuals who wanted to share the link to the survey. The study was also featured in national and local newspapers.

### Sample

Norwegian citizens aged 18 years or older were invited to participate. There were no exclusion criteria.

### Measures

Sociodemographic and health-related data were collected as self-report measures via the web-based survey. The survey employed several measures identical to the ones used in the Norwegian population health survey (the NORPOP study), which was conducted as a postal survey in 2014–2015 (18–21). New questions pertaining to the possible concerns and responses to the COVID-19 pandemic were developed by the research group at the time of the pandemic outbreak.

#### Sociodemographic Variables

Data were collected for age group (18–29 years, 30–39 years, 40–49 years, 50–59 years, 60–69 years, and 70 years or older) gender (male/female), highest completed education level (elementary school, high school, <4 years of higher education, and 4 years of higher education or more), employment status (working/in education vs. not), cohabitation status (living with spouse or partner vs. not), and living with children (living with children under the age of 18 years vs. not). Size of place of residence was categorized as <200 inhabitants, 200–19.999 inhabitants, 20.000–99.999 inhabitants, and 100.000 inhabitants or more. In Norway, a significant part of the population lives in rural areas. The categories were made considering that about 20–30% of the population belong to each of the categories, and the NORPOP study (18–21) used the same categories.

#### Substance Use

We used the phrase: “Use of alcohol and addictive drugs and pharmaceuticals: have you used any of these?” Below was a list containing alcohol, cannabis, sedatives, and painkillers/opioids, with examples provided for sedatives and painkillers/opioids. Response options were “no,” “sometimes,” “weekly,” “daily,” and “several times daily.” Participants who reported that they used alcohol daily were classified as daily drinkers. Participants who reported that they (had) used cannabis, sedatives or painkillers/opioids “sometimes” or more often, were classified as “sometimes, weekly or daily” (S/W/D) users of the relevant substance.

#### Mental Health Problems

We used the phrase: “Below is a list of health problems. Do you have, or have you had, any of these?” Among the listed problems were anxiety, depression, insomnia, and suicide thoughts. The response alternatives were “no,” “yes previously, but not during the last month” and “yes, during the last month.” Those who confirmed having one or more of the listed health problems during the last month were classified as currently having the relevant mental health problems.

#### Problems Related to the Pandemic

Relating to the COVID-19 situation, participants were asked to respond “yes” or “no” to the following questions: (a) “Are you suffering, or do you think you will be suffering, economic loss?”, (b) “Have you been in quarantine or in isolation due to the corona virus?”, (c) “Are you at risk of experiencing complications from COVID-19?”, and (d) “Do you have friends or close family that you worry about?” Participants who indicated “yes” on any of these questions were classified as “experiencing/expecting economic loss,” “experienced quarantine/isolation,” “risk of complication,” and/or “worry about friends/family,” respectively.

### Statistical Analyses

Frequencies and proportions (%) were calculated for all categorical variables, and all were cross tabulated with the occurrence of daily alcohol use, and with sometimes/weekly/daily use of cannabis, sedatives, and painkillers. Single and multiple logistic regression analyses were performed to assess associations between sociodemographic variables, mental health problems and COVID-19 related problems, and the use of substances. In the multiple logistic regression analyses, all independent variables were entered together in order to cancel out the effects of covariation between the independent variables. *Post-hoc* interaction analyses were performed for alcohol use to assess whether significant associations were dependent on levels of sociodemographic variables showing skewed distributions (i.e., age group, gender, education, and size of place of residence). Interaction terms were included separately in a second step of the analysis, while adjusting for sociodemographic variables, mental health problems and COVID-19 related problems. For alcohol, we distinguished between daily use (1) vs. less frequent or no use (0). For cannabis, sedatives, and painkillers, we distinguished between sometimes/weekly/daily use (1) vs. no use (0). Odds ratio (OR) with 95% confidence interval (CI) was reported. IBM SPSS Statistics version 26 (22) was used for statistical analyses, and the significance level was set at 5%.

### Ethics

The questionnaires were answered anonymously. Ethical approval for conducting the study was granted from the Regional Committee for Medical and Healthcare Ethics (REK no. 130447).

## Results

### Sample Characteristics

The sociodemographic characteristics of the sample is displayed in Table 1. More than half of the sample was under 40 years of age, and the number of participants was lower in the higher age groups. A majority were women (85.0%), had higher education (75.5%) and were employed or in education (81.0%). Current anxiety and depression were reported by 17.1 and 12.5%, respectively, while 31.8% reported insomnia. A smaller proportion (3.6%) reported having had suicide thoughts during the last month, while the larger proportion (61.7%) reported having none of the listed mental health problems. With regards to COVID-19, 25.3% expected to suffer financial loss, 28.3% had been quarantined or in isolation and 23.4% reported to be at risk of experiencing complications if contracting the coronavirus. The large majority (83.9%) were worried about someone close to them.

**Table 1:** Sociodemographic characteristics of the sample (*n* = 4,527), among daily users of alcohol (*n* = 138) and among sometimes/weekly/daily users of cannabis (*n* = 139), sedatives (*n* = 241), and painkillers (*n* = 579).

**Characteristics**	**Total sample**	**Daily alcohol**	**Sometimes/weekly/daily**	**Sometimes/weekly/daily**	**Sometimes/weekly/daily**
			**cannabis**	**sedatives**	**painkillers**
**Age group**	***n*** **(%)**	***n*** **(%)**	***n*** **(%)**	***n*** **(%)**	***n*** **(%)**
18–29	1156 (25.5)	20 (1.7)	70 (6.1)	61 (5.3)	121 (10.5)
30–39	1220 (26.9)	32 (2.6)	34 (2.8)	55 (4.5)	149 (12.2)
40–49	931 (20.6)	30 (3.2)	23 (2.5)	46 (4.9)	131 (14.1)
50–59	766 (16.9)	29 (3.2)	7 (0.9)	45 (5.9)	116 (15.1)
60–69	354 (7.8)	17 (4.8)	3 (0.8)	23 (6.5)	39 (11.0)
70 or above	100 (2.2)	10 (10.0)	2 (2.0)	11 (11.0)	23 (23.0)
**Gender**^**a**^					
Male	659 (14.6)	37 (5.6)	52 (7.9)	39 (5.9)	98 (14.9)
Female	3850 (85.0)	101 (2.6)	87 (2.3)	200 (5.2)	479 (12.4)
**Highest completed education**^**b**^					
Elementary school	591 (13.1)	13 (2.2)	29 (4.9)	57 (9.6)	126 (21.3)
High school	514 (11.4)	14 (2.7)	23 (4.5)	33 (6.4)	84 (16.3)
Higher education <4 years	1376 (30.4)	45 (3.3)	38 (2.8)	66 (4.8)	167 (12.1)
Higher education ≥4 years	2041 (45.1)	66 (3.2)	48 (2.4)	84 (4.1)	202 (9.9)
**Employment**					
Employed or in education	3667 (81.0)	100 (2.7)	103 (2.8)	141 (3.8)	396 (10.8)
Not employed and not in education	860 (19.0)	38 (4.4)	36 (4.2)	100 (11.6)	183 (21.3)
**Cohabitation status**					
Living with spouse or partner	2714 (60.0)	86 (3.2)	61 (2.2)	126 (4.6)	354 (13.0)
Not living with spouse or partner	1813 (40.0)	52 (2.9)	78 (4.3)	115 (6.3)	225 (12.4)
**Living with children** ** <18 years**					
Living with children	1547 (34.2)	35 (2.3)	30 (1.9)	60 (3.9)	194 (12.5)
Not living with children	2980 (65.8)	103 (3.5)	109 (3.7)	181 (6.1)	385 (12.9)
**Size of place of residence**^**c**^					
Rural (<200 inhabitants)	187 (4.1)	3 (1.6)	6 (3.2)	8 (4.3)	36 (19.3)
Village (200–19,999 inhabitants)	1141 (25.2)	27 (2.4)	27 (2.4)	76 (6.7)	161 (14.1)
Town (20,000–99,999 inhabitants)	1091 (24.1)	29 (2.7)	24 (2.2)	57 (5.2)	142 (13.0)
City (>100,000 inhabitants)	2098 (46.3)	79 (3.8)	81 (3.9)	99 (4.7)	238 (11.3)

a *Eighteen participants (0.4%) did not state gender*.

b *Five participants (0.1%) did not state education level*.

c *Ten participants (0.2%) did not state size of place of residence*.

### Associations Between Use of Alcohol and Problems Related to Mental Health and the COVID-19 Situation

As shown from the multiple logistic regression analysis in Table 2, the odds of using alcohol daily were higher for those of higher age (OR: 1.31, *p* < 0.001) and among those with higher education (OR: 1.61, *p* < 0.05), and lower among women than men (OR: 0.50, *p* < 0.01). Daily use of alcohol was also associated with depression (OR: 3.40, *p* < 0.001) and with expecting financial loss in relation to the COVID-19 outbreak (OR: 1.66, *p* < 0.01).

**Table 2:** Associations with daily use of alcohol.

**Independent variables**	**Crude**			**Adjusted**		
	**OR**	**95%CI**	***P***	**OR**	**95%CI**	***p***
Age group	1.33	1.18–1.50	<0.001	1.31	1.14–1.51	<0.001
Female gender	0.45	0.31–0.66	<0.001	0.50	0.34–0.75	<0.01
Higher education	1.34	0.88–2.06	0.18	1.61	1.03–2.52	<0.05
Employment	0.60	0.41–0.88	<0.05	0.98	0.63–1.52	0.93
Anxiety	1.43	0.95–2.14	0.09	0.69	0.41–1.18	0.17
Depression	3.35	2.31–4.85	<0.001	3.40	2.06–5.62	<0.001
Insomnia	1.68	1.19–2.37	<0.01	1.18	0.78–1.78	0.43
Suicide thoughts	2.98	1.64–5.39	<0.001	1.71	0.86–3.38	0.12
Economic loss	1.82	1.28–2.59	<0.01	1.66	1.15–2.41	<0.01
Quarantine/isolation	1.33	0.93–1.90	0.12	1.41	0.98–2.03	0.07
Risk of complications	1.96	1.38–2.79	<0.001	1.31	0.88–1.95	0.19
Model fit				*p* < 0.001		
Cox Snell (Nagelkerke)				2.0% (8.5%)		

*Dependent variable is daily use of alcohol. Age group is 10-year intervals. Employment is being employed or in education. Anxiety, depression, insomnia, and suicide thoughts are having the problem during the last month. Economic loss is expecting the COVID-19 situation to cause personal economic loss. Risk of complications is self-reported risk of complications in the case of contracting the coronavirus*.

The *post-hoc* analyses revealed a significant interaction between age group and depression (*p* = 0.02). Cross tabulating depression and daily alcohol use for each of the age groups revealed a significant association for participants aged 18–29 years (ϕ = 0.15, *p* < 0.001), 30–39 years (ϕ = 0.15, *p* < 0.001), and 40–49 years (ϕ = 0.13, *p* < 0.001), while the association was not significant for participants in the older age groups. Thus, depression was more strongly related to daily alcohol use in the younger age groups. None of the other tested interaction terms were found to be statistically significant.

### Associations Between Use of Cannabis and Problems Related to Mental Health and the COVID-19 Situation

As shown from the multiple logistic regression analysis in Table 3, the odds of using cannabis were lower for those of higher age (OR: 0.53, *p* < 0.001) and among women (OR: 0.19, *p* < 0.001). The odds of using cannabis were higher among those who expected financial loss in relation to the COVID-19 outbreak (OR: 1.62, *p* < 0.05).

**Table 3:** Associations with use of cannabis.

**Independent variables**	**Crude**			**Adjusted**		
	**OR**	**95%CI**	***P***	**OR**	**95%CI**	***p***
Age group	0.60	0.51–0.71	<0.001	0.53	0.44–0.63	<0.001
Female gender	0.27	0.19–0.38	<0.001	0.19	0.13–0.28	<0.001
Higher education	0.52	0.37–0.74	<0.001	0.71	0.49–1.03	0.07
Employment	0.66	0.45–0.97	<0.05	0.71	0.46–1.11	0.13
Anxiety	1.48	0.99–2.21	0.06	0.96	0.58–1.59	0.87
Depression	1.56	1.00–2.42	<0.05	1.06	0.60–1.89	0.85
Insomnia	1.61	1.14–2.27	<0.01	1.39	0.93–2.07	0.11
Suicide thoughts	1.46	0.67–3.17	0.34	0.75	0.31–1.80	0.52
Economic loss	2.17	1.54–3.06	<0.001	1.62	1.12–2.34	<0.05
Quarantine/isolation	1.36	0.95–1.94	0.09	1.18	0.82–1.71	0.37
Risk of complications	0.94	0.63–1.41	0.76	1.25	0.80–1.95	0.33
Model fit				*p* < 0.001		
Cox Snell (Nagelkerke)				3.1% (12.8%)		

*Dependent variable is use of cannabis sometimes, weekly or daily. Age group is 10-year intervals. Employment is being employed or in education. Anxiety, depression, insomnia, and suicide thoughts are having the problem during the last month. Economic loss is expecting the COVID-19 situation to cause personal economic loss. Risk of complications is self-reported risk of complications in the case of contracting the coronavirus*.

### Associations Between Use of Sedatives and Problems Related to Mental Health and the COVID-19 Situation

As shown from the multiple logistic regression analysis in Table 4, the odds of using sedatives were higher for those of higher age (OR: 1.17, *p* < 0.01) and lower among those having employment (OR: 0.58, *p* < 0.01). Use of sedatives was also associated with anxiety (OR: 4.76, *p* < 0.001), depression (OR: 1.64, *p* < 0.01), and insomnia (OR: 2.15, *p* < 0.001).

**Table 4:** Associations with use of sedatives.

**Independent variables**	**Crude**			**Adjusted**		
	**OR**	**95%CI**	***P***	**OR**	**95%CI**	***p***
Age group	1.11	1.01–1.21	<0.05	1.17	1.05–1.31	<0.01
Female gender	0.87	0.61–1.23	0.43	0.76	0.52–1.13	0.17
Higher education	0.52	0.39–0.68	<0.001	0.83	0.62–1.13	0.24
Employment	0.30	0.23–0.40	<0.001	0.58	0.42–0.80	<0.01
Anxiety	8.64	6.59–11.34	<0.001	4.76	3.36–6.74	<0.001
Depression	6.24	4.75–8.19	<0.001	1.64	1.15–2.34	<0.01
Insomnia	5.14	3.89–6.80	<0.001	2.15	1.54–2.99	<0.001
Suicide thoughts	5.57	3.74–8.31	<0.001	1.33	0.83–2.14	0.23
Economic loss	1.56	1.18–2.05	<0.01	0.99	0.72–1.34	0.93
Quarantine/isolation	1.00	0.75–1.34	0.99	0.85	0.62–1.17	0.32
Risk of complications	2.29	1.75–2.99	<0.001	1.30	0.95–1.79	0.10
Model fit				*p* < 0.001		
Cox Snell (Nagelkerke)				7.3% (21.3%)		

*Dependent variable is use of sedatives sometimes, weekly or daily. Age group is 10-year intervals. Employment is being employed or in education. Anxiety, depression, insomnia, and suicide thoughts are having the problem during the last month. Economic loss is expecting the COVID-19 situation to cause personal economic loss. Risk of complications is self-reported risk of complications in the case of contracting the coronavirus*.

### Associations Between Use of Painkillers and Problems Related to Mental Health and the COVID-19 Situation

As shown from the multiple logistic regression analysis in Table 5, the odds of using painkillers were lower for those with higher education (OR: 0.64, *p* < 0.001) and those with employment (OR: 0.66, *p* < 0.001). Use of painkillers was also associated with insomnia (OR: 1.48, *p* < 0.001) and reporting risk of complications if contracting the coronavirus (OR: 1.57, *p* < 0.001).

**Table 5:** Associations with use of painkillers.

**Independent variables**	**Crude**			**Adjusted**		
	**OR**	**95%CI**	***P***	**OR**	**95%CI**	***p***
Age group	1.12	1.05–1.19	<0.01	1.05	0.98–1.13	0.18
Female gender	0.81	0.64–1.03	0.08	0.86	0.67–1.10	0.22
Higher education	0.52	0.43–0.62	<0.001	0.64	0.52–0.78	<0.001
Employment	0.45	0.37–0.55	<0.001	0.66	0.53–0.82	<0.001
Anxiety	1.58	1.28–1.94	<0.001	1.01	0.77–1.32	0.95
Depression	1.85	1.47–2.32	<0.001	1.24	0.92–1.66	0.15
Insomnia	1.84	1.54–2.19	<0.001	1.48	1.20–1.81	<0.001
Suicide thoughts	1.86	1.26–2.75	<0.01	1.09	0.70–1.70	0.72
Economic loss	1.36	1.12–1.64	<0.01	1.13	0.92–1.39	0.23
Quarantine/isolation	1.13	0.93–1.37	0.21	1.09	0.89–1.32	0.41
Risk of complications	2.05	1.70–2.46	<0.001	1.57	1.27–1.93	<0.001
Model fit				*p* < 0.001		
Cox Snell (Nagelkerke)				3.2% (6.0%)		

*Dependent variable is use of painkillers sometimes, weekly or daily. Age group is 10-year intervals. Employment is being employed or in education. Anxiety, depression, insomnia, and suicide thoughts are having the problem during the last month. Economic loss is expecting the COVID-19 situation to cause personal economic loss. Risk of complications is self-reported risk of complications in the case of contracting the coronavirus*.

## Discussion

This study examined use of alcohol and addictive drugs in the Norwegian population during the COVID-19 outbreak and examined the substance use in association with mental health problems and problems related to the pandemic. The occurrence of mental health problems was found to be more important for an understanding of the use of alcohol and addictive drugs during the COVID-19 outbreak, compared to specific pandemic-related worries.

Daily use of alcohol was associated with expecting financial loss in relation to the COVID-19 outbreak. Some people may have felt that their lives had been turned upside down regardless of the consequence for their personal economy, while others have lost their jobs, or they lived in constant fear of losing it (3). The expectance of financial loss may be frequently occurring among people employed with private sector jobs that were strongly affected by market fluctuations, which may translate into an increased risk of losing their job. Many businesses in Norway were temporarily closed at the onset of COVID-19, resulting in a rapid increase in unemployment rates (4). The consequences were particularly severe for people employed in the transportation and tourism industries, for which unemployment rates were twice as high compared to other industries (4). Although one might assume a relationship between the expectance of financial loss and depression, the results substantiated an association between expecting financial loss and daily use of alcohol that was independent of depression.

Adjusted for all variables, daily use of alcohol retained its association with depression. The detected association between alcohol use and depression is in line with a range of studies (23), including a recent Norwegian population study in which having anxiety or depression was associated with daily alcohol use (24). Thus, use of alcohol appears to be relatively independent from external circumstances. As demonstrated by Ertl et al. (25), alcohol use in the context of mental health problems may be linked with relief-oriented motives for drinking, as opposed to reward-oriented motives. In their study, more psychopathology was associated with relief-oriented drinking motives. It is possible that relief-oriented drinking may increase during crises as many people struggle with stress reactions to the crisis. The results from the *post-hoc* interaction analyses suggest that this may more often be the case among people in the younger age groups. A study from Finland showed that more psychological symptoms predicted a pattern of heavy drinking from adolescence to midlife (26). Although the cross-sectional design of this study prohibits concluding about the direction of the association, depression appears to be consistently linked with heavy and frequent use of alcohol during the COVID-19 outbreak, as shown in other countries alike (13). Lastly, we noted the association between higher education levels and higher odds of daily use of alcohol. While this indicates that a high-frequent drinking pattern is more common among those with higher education, it does not necessarily point toward more alcohol-related problems in this group.

Use of cannabis was associated with expecting financial loss in relation to the COVID-19 outbreak. Cannabis use was also associated with younger age, a finding which is in line with previous Norwegian population studies (27). Generally, young people are often employed in part-time jobs while undergoing education—in Norway, one in three full-time students have part time jobs (28). Moreover, they often have jobs in the retail and service industries such as shops and restaurants, which might be particularly affected by crises such as the COVID-19 outbreak. Thus, people of young age are generally in a vulnerable economic situation which may become even worse with the potential loss of a job.

Use of sedatives was associated with anxiety, depression, and insomnia. This result appears to reflect that sedatives are commonly used pharmaceuticals for mental disorders in general (29). It is also consistent with the findings in a recent general population study from Norway (15), demonstrating higher relative risk of a wide range of disorders, including anxiety, depression and insomnia, for people who had used sedatives sometimes or more often.

Use of painkillers was associated with insomnia and reporting risk of complications if contracting the coronavirus. Sleep and pain have been shown to have a bidirectional relationship—sleep problems can be caused by pain, while better sleep can reduce pain (30). For people experiencing pain, the use of painkillers can therefore be a logical means to regaining sleep. Use of painkillers have also been shown to be associated with higher risk of a wide range of diseases, including pulmonary disease and cancer (15). As these diseases are often painful (31, 32) and constitute increased risk of fatal outcome if the person is exposed to COVID-19 infection, the occurrence of these and similar pain-inducing diseases may contribute to explain the association between perceived risk of complications and use of painkillers.

### Study Limitations

The cross-sectional survey design precludes us from establishing causal relationships between the variables under study; the study is therefore limited in its mere detection of statistical associations between mental health problems and problems relating to the COVID-19, and the use of alcohol and addictive drugs. To overcome some of the limitations related to a cross-sectional study design, future studies may include follow-up assessments that will allow for studying how the use of alcohol and drugs develops over time, and how their use may be related to a range of specific exposures.

While a range of variables were used as possible predictors of substance use, other variables—not accounted for in this study—might have added to the explanatory power of the statistical model. Such variables may include confinement at home, working from home, spending more time together at home, home conflicts, and having children at home during daytime. Possibly, the consumption of substances would be related to these situations. Alternatively, these situations may increase the risk of mental health problems, which in turn was found to be related to use of substances.

The use of standard mental health measurements might have been a preferred option to our use of single-item scales, and future studies may include the use of standardized measures such as the Alcohol Use Disorder Identification Test (AUDIT) (33, 34). The recently developed Fear of COVID-19 Scale has shown to be promising (35), and the Norwegian version of the scale may also be serviceable (36). However, standard measurements are generally longer and place a larger burden on participants, especially when used in context of a larger survey. Moreover, studies have demonstrated that the use of single-item measures can be a valid and reliable option for measuring mental health phenomena (37–40). Similarly, with regards to alcohol use, measuring the quantity of drinking in addition to the frequency of drinking may have provided a more comprehensive estimate of risky drinking. Nonetheless, evidence suggests that high-frequent drinking is associated with increased health risk, even in the case of moderate consumption (41, 42).

The recruitment strategy was based on disseminating the link to the survey via various social media, and the strategy makes generalizing the results to the general population impossible due to selection bias. In fact, the sample was dominated by young, urban, and highly educated persons, and the vast majority were female. Among the general population in Norway, a significant part lives in rural areas, but this rural population was underrepresented in this study. Therefore, one should be cautious when comparing prevalence rates related to the use of alcohol and addictive drugs to previously established prevalence rates in the general population. In comparison to previously established prevalence rates (15, 24), it appears that the rate of daily alcohol use is similar, whereas the rates of “sometimes/weekly/daily” use of cannabis, sedatives, and painkillers are lower in this study. However, it has been argued that skewed samples are more prone to affect prevalence rates of substance use, while less prone to affect associations between predictors and substance use outcomes (43, 44). The *post-hoc* interaction analyses conducted with regards to alcohol use in this study were largely in line with this view. The relatively large sample size and the possibility to compare the results from the early phase of the COVID-19 outbreak with a previous general population study (NORPOP) are strengths of the study. However, we stress that the study was conducted exclusively with participants living in Norway, which makes it impossible to transfer the conclusions of the study to other countries. Similar studies conducted in other countries and settings are therefore warranted.

## Conclusions

Compared to specific pandemic-related worries, the occurrence of mental health problems was found to be more important for the use of alcohol and addictive drugs during the early stage of the COVID-19 outbreak in Norway. Depression was associated with daily use of alcohol, while several mental health problems including depression, anxiety, and insomnia were associated with use of sedatives. Worries specifically related to the COVID-19 outbreak were of less importance in relation to the use of alcohol and addictive drugs. While the results of a population survey do not warrant direct application to individual clients with alcohol or drug problems, the study indicates that mental health problems constitute an important context for the use of alcohol and addictive drugs during the pandemic. Public health initiatives aimed at reducing harmful use of alcohol and drugs should therefore consider the occurrence of mental health problems in the targeted population. Conversely, increased incidence of mental health problems in a population may warrant further assessment of service needs related to substance use.

## Data Availability Statement

The raw data supporting the conclusions of this article will be made available by the authors, without undue reservation, upon completion of the research project.

## Ethics Statement

The studies involving human participants were reviewed and approved by Regional Committee for Medical and Healthcare Ethics. Written informed consent for participation was not required for this study in accordance with the national legislation and the institutional requirements.

## Author Contributions

TB, TH, IS-B, ØE, LS, and TG: conceptualization, methodology, validation, investigation, and writing—review and editing. TB: formal analysis, data curation, writing—original draft preparation, visualization. TG: project administration. All authors have read and agreed to the published version of the manuscript.

## Conflict of Interest

The authors declare that the research was conducted in the absence of any commercial or financial relationships that could be construed as a potential conflict of interest.
